# Design and Implementation of an Infrared Radiant Source for Humidity Testing

**DOI:** 10.3390/s18093088

**Published:** 2018-09-13

**Authors:** Hong Zhang, Chuansheng Wang, Xiaorui Li, Boyan Sun, Dong Jiang

**Affiliations:** School of Computer Science and Technology, Harbin University of Science and Technology, 52 Xuefu Road, Harbin 150080, China; wangchuansheng994@163.com (C.W.); 13682088813@163.com (X.L.); 15754509280@163.com (B.S.); wdyu2004@163.com (D.J.)

**Keywords:** infrared radiant source, Monte Carlo method, emissivity, calibration, humidity

## Abstract

A novel way to measure humidity through testing the emissivity of an area radiant source is presented in this paper. The method can be applied in the environment at near room temperature (5~95 °C) across the relative humidity (RH) range of 20~90% RH. The source, with a grooved radiant surface, works in the far infrared wavelength band of 8~12 μm. The Monte-Carlo model for thermal radiation was set up to analyze the V-grooved radiant surface. Heat pipe technology is used to maintain an isothermal radiant surface. The fuzzy-PID control method was adopted to solve the problems of intense heat inertia and being easily interfered by the environment. This enabled the system to be used robustly across a large temperature range with high precision. The experimental results tested with a scanning radiant thermometer showed that the radiant source can provide a uniform thermal radiation capable of satisfying the requirements of humidity testing. The calibration method for the radiant source for humidity was explored, which is available for testing humidity.

## 1. Introduction

Compared to traditional humidity measurement methods, innovative electronic testing methods involving humidity sensors such as hygristors and humicaps are the current research direction. Even though electric methods have fast responses, they often lack stability and their accuracy is improved little. Widely available commercial humidity sensors composed of humicaps use embedded microprocessors, such as the DHT11 with ±5% RH precision, and 1% RH resolution which are convenient to use. Because humidity is often mingled together with temperature, the precision and the humidity measurement range are easily affected by the temperature. Humidity, which reflects the degree of dryness of the atmosphere, is an important variable that is extensively tested in agriculture, industry, hospital and warehouse. Some sensors with new materials possessing resistive and capacitive features have been explored, which include a sulfonated polycarbonate resistive humidity sensor [1], polyimide-based capacitive humidity sensor [2], a high-performance capacitive humidity sensor with novel electrode and polyimide layer capacitive humidity sensors [3], and some sensors with improved sensing properties whose response and recovery times are 14.5 s and 34.27 s, respectively, for humidity levels between 33% RH and 95% RH at 102 Hz. [4]. These kinds of humidity sensors often have long response times. Most studies focus on material and processing innovations to increase the humidity testing precision. There have been few breakthroughs in hygristors because these are easily interfered by the environment. Novel ways using new effects such as optical properties present approaches to measure humidity [5]. Because humidity is a factor that affects the radiation measurement, humidity can be measured indirectly by testing radiation changes [6,7].

As a standard radiant source, a blackbody is usually adopted for calibrating infrared instruments such as pyrometers and radiant thermometers. Industrial blackbodies ranging from −50~2500 °C have been developed by the National Research Council (NRC) of Canada in order to calibrate optical testing devices [8]. A high-spatial-resolution multi-spectral imager (ASTER) on the first platform (Terra) of NASA’s Earth Observing System requires a blackbody radiant source on a satellite for calibration purposes [9]. Traditional blackbody cavities evaluated by the Bedford methods [10] usually possess symmetrical shapes with small apertures, which makes them suitable for the high temperature range case, but not for the case of near room temperature range measurements. Minimum Resolvable Temperature Difference (MRTD) sensitivity requires that a radiant source working in the far infrared scope should be an area source which can provide a stable radiant flux with high uniformity. As to environmental humidity measurement, according to MRTD, an area radiant source ranging from 5~95 °C in the wavelength bands of 8~12 µm, is required. Under a certain temperature, a source should emit a stable radiation which is monitored by a radiometer. In order to enhance the effective emissivity of a radiant surface, its surface is often processed into grooves or mini holes. The radiant surface of the source possesses concentric V-grooves which can increase the effective emissivity.

Among statistical evaluation methods, Monte Carlo methods have been widely applied in optical radiometry and blackbody cavity analysis [11]. Monte Carlo methods possess advantages which are greater than exactitude methods in complex radiant characteristic analysis. Therefore, the Monte Carlo method is adopted to analyze the effective emissivity of the radiant source. After a theoretical analysis on the distribution of the effective emissivity of the radiant surface, the source structure was constructed. Heat pipe technology keeps the source isothermal and the temperature control system ensures that the source is stable at a certain temperature. The radiant source has a broad applications in various fields, such as infrared imaging, infrared measurement and humidity test.

Although there are tens of ways to measure humidity, among which the most traditional methods are the dry and wet bulb thermometer whose precision is lower compared with modern electronic methods, most of these ways are not satisfactory in terms of precision and stability [7]. Capacitor sensors which possess fast response advantages are employed to test humidity, but their measuring precision is easily affected by electromagnetic interference. Besides, humidity testing is often affected by the environmental temperature, which is a factor that makes humidity sensors’ precision not be high. Humidity testing through radiation possesses advantages of fast response and robustness with high precision. Our research on an infrared source which has highly sensitivity for humidity may provide an improved way to measure humidity.

## 2. Analysis on Characteristics of Radiant Surface

The Monte-Carlo method was utilized for analyzing the radiant surface with concentric V-grooves. Assuming that the surface is diffuse (Lambertian), the calculation on its luminance follows Lambert’s cosine Law. The Monte-Carlo Model of thermal radiation was set up. A Monte-Carlo simulation is implemented through random sampling that is based on probability models whose deduction methods are based upon actual physical models. Exactitude methods like the Bedford method are just suitable to calculate simple symmetric cavity shapes. Although Monte-Carlo methods are flexible enough to be used for complicated cases, they are often regarded as unreliable methods with low precision. The effective emissivity of a cone was calculated by both the Monte Carlo method and the Bedford method, respectively. By comparing the results from the both methods, the correctness of the Monte Carlo method was proven. 

### 2.1. Monte-Carlo Model in Thermal Radiation

**Figure 1:**
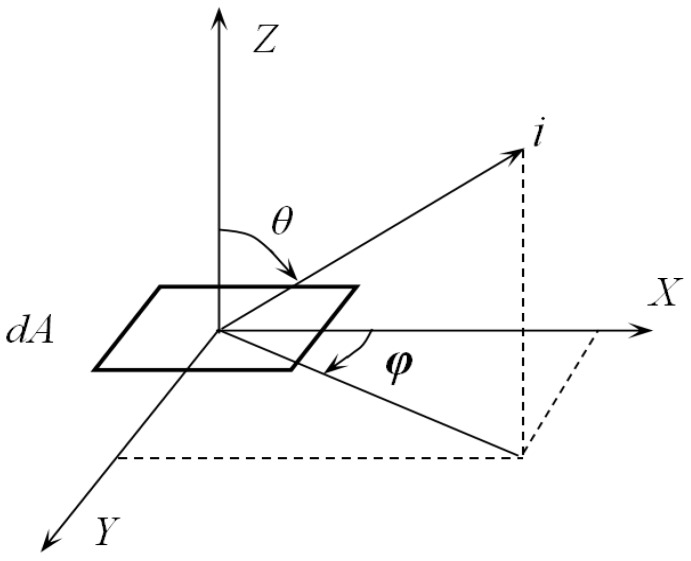
Direction of an emitted beam.

The idea of a Monte-Carlo method is to set up a probability model or stochastic process whose parameters are equal to the solution of the problem to investigate, then to calculate the statistical characteristics of the required parameters through sampling, and the solution can be solved based on a vast number of observations. In thermal radiation calculations, local temperature and radiation fluxes are usually involved. The process of thermal radiation exchange is regarded as the movement of discrete energy beams. In this way, the local radiation flux can be obtained by calculating the number of beams that reach the local surface per unit time. These beams are deemed to consist of particles with a certain amount of energy. If the energy of each beam is equal, the local energy flux can be obtained by multiplying the number of beams arriving by the energy of each bundle of light per unit time per unit area. The direction of a transmitting beam *i* is determined by the direction angles *θ*, *φ* which are obtained by random sampling, as shown in Figure 1. *dA* is the area of a tiny piece. The radiation from *dA* has different intensity along with directions. For a diffuse surface, the emssivity of a surface is independent of the azimuth angle *φ*, but dependent on *θ*. The monochromatic emissivity of the surface is independent of the azimuth angle *φ* (The word monochromatic means the emissivity is under a certain wavelength *λ*, it is correspondent to the word ‘total’ that covers the whole spectrum, which means the wavelength *λ* is from 0~∞), and the total emission energy per unit time is as below:

(1) $$E=\varepsilon \sigma T^{4}dA$$

where *ε* is the material emissivity, *T* is the temperature of *dA* Kelvin, *σ* is Boltzmann’s constant.

**Figure 2:**
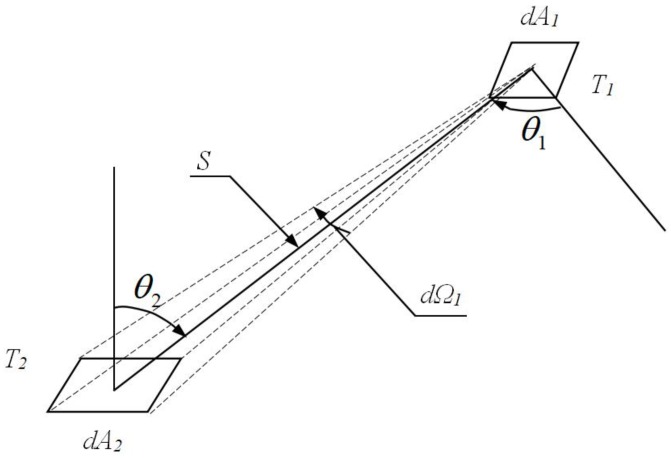
Radiant exchanging relation between two tiny surfaces.

Assuming that there are two tiny pieces of blackbody *dA*_1_, *dA*_2_, and there exists radiation exchange between both the tiny pieces, then the angle coefficient *F*_*d*1__*−d*2_ is defined by the ratio of the energy radiated out from *dA*_1_ reached *dA*_2_ to the total energy radiation of *dA*_1_, as shown in Figure 2.

**Figure 3:**
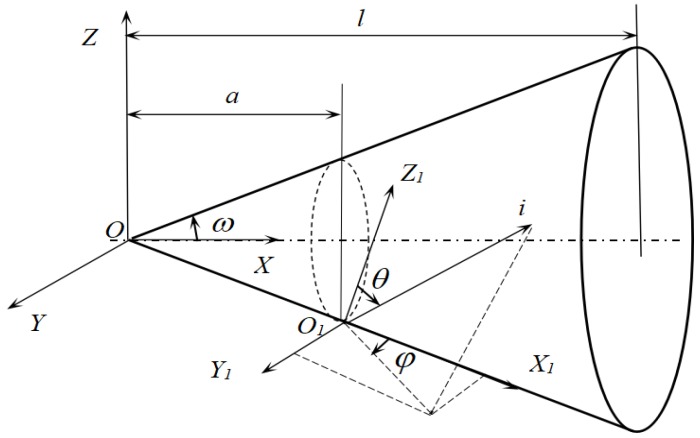
Light ray tracing in a cone cavity.

When a Monte Carlo method is used in analyzing the characteristics of a blackbody cavity (as shown in Figure 3), the radiation of the light point *i* can be treated as two components, one is *EF_i_* which radiates out of the aperture, the other is *E*(1 − *F_i_*) which goes to the wall at where it may be absorbed or reflected. If it is reflected then it is divided into two parts again which are *E*(1 − *F_i_*)*F_i_*_1_ and *E*(1 − *F_i_*)(1 − *F_i_*_1_), where *F_i_*_1_ denotes the angle factor between the point at where the beam from *i* is reflected first time. Assuming *F_ij_* that denotes the angle factor between the point where the ray from *i* reflected at the time *j*, a beam of light can be traced in this way. When all the light points have been manipulated, the radiant flux out of the cavity and the effective emissivity are obtained [12]. However, for the V-groove concentric circles surface, the above model is not appropriate. The Monte-Carlo model is used to simulate the real physical model [13], in which radiant flux is coming from light points which are distributed uniformly along the surface. Each beam of emitting lights possesses the same amount of energy which is the total radiant energy of the point (it is an infinitesimal area *dA*), as shown in Equation (1). A light point’s position is *a* that can be determined according to the geometry probability, and a ray’s direction is determined by zenith *θ* and azimuthal *φ*. The event that a ray is reflected or absorbed is treated as a random variable [14]. If a surface is diffuse, the probability model of the random sampling *θ* is as follows:

(2) $$P(\theta )=\int_{0}^{\theta }2\sin\theta \cos\theta d\theta =\sin^{2}\theta \;$$

where *r* is random number. *θ* can be determined by generating *r*, *φ* is determined by *φ* = 2*πr*. All beams are traced thoroughly, and the evaluation of effective emissivity is totally based on the statistics of a tremendous number of samples, but simulated results are significantly affected by many factors such as the correctness of a Monte-Carlo model, the way light ray tracing is judged, sampling number, sampling method and the uniformity of the random number generator, etc.

### 2.2. Monte-Carlo Simulation for a Cone Cavity

To calculate the effective emissivity of a cone cavity by the Monte Carlo method, the simulation procedure is as follows: the first step is to determine the position of a light point, *a*, (as shown in Figure 3) according to the area probability:

(3) $$P(a)=\frac{\pi a^{2}\sin\omega /\cos\omega^{2}}{A_{cone}}\;$$

where *A_cone_* is the cone area, as a continue variable *a*, its random sampling is *r* = *P*(*a*), where *r* is a random number, *a* is determined as follows:

(4) $$a=\sqrt{\frac{rA_{cone}\cos^{2}\omega }{\pi \sin\omega }}\;0\;\leq \;a\;\leq \;l$$

The second step is to determine the beam’s direction (*θ, φ*), *θ* random sampling is similar as *a*, according to Equation (2), the direct sampling method is as: $\theta =\sin^{-1}\sqrt{r}$, φ=2πr.

In order to reduce the computing time, *θ* can be obtained by the rejection sampling method as follows: $\theta =r_{1}\pi /2$ when $r_{2}<\sin\pi r_{1}$. *r*_1_, *r*_2_ are random numbers.

The third step is to trace the beam, to judge where it goes, if it flies out of the cone, the accumulated radiant energy out *E_out_*, if it is still in the cone, to judge that if it is absorbed or reflected, the cone equation in coordinate OXYZ is:

(5) $$x^{2}tg^{2}\omega =y^{2}+z^{2}\;$$

The ray’ equation in coordinates O1X1Y1Z1:

(6) $$\{\begin{array}{c} z_{1}^{2}=ctg^{2}\theta (x_{1}^{2}+y_{1}^{2})\\ y_{1}=x_{1}tg\omega \end{array}\;$$

The transformation between OXYZ and O1X1Y1Z1 is as shown below:

(7) $$\{\begin{array}{c} x=x_{1}\cos\omega +z_{1}\sin\omega +a\\ z=-x_{1}\sin\omega +z_{1}\cos\omega -atg\omega \end{array}\;$$

The crossing point of the ray and the cone is obtained from Equations (5) and (6), its coordinate in OXYZ is *x* (according to Equation (7)), if x>0 and x<1, the ray is still in the cone, otherwise it flies out, then the total energy *E_out_* accumulates, then we go back to the first step.

If the ray is still in the cone, and when r≤ε, it is absorbed, and the program flow returns back to the original procedure to generate another new light point, otherwise (r>ε) the ray is reflected. To take *x* as *a*, it goes back to the second step. When all the beams are traced, the effective emissivity $\varepsilon_{a}P(0)$ (0 represents the center cone) will be obtained as follows, $A_{ap}$ is the area of the cone aperture:

(8) $$\varepsilon_{a}P(0)=\frac{E_{out}}{\sigma T^{4}A_{aP}}\;$$

### 2.3. Bedford Method for a Cone Cavity

**Figure 4:**
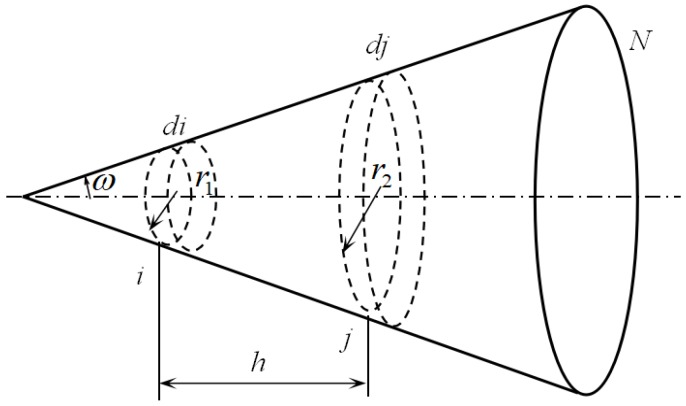
Radiation exchange in a cone cavity.

The cone is divided into *N* segments along the axis. *i* represents the position and also the disk at the position *i*, *di* means the ring at *i*, $F_{i-j}$ is the angle factor between disk i and disk j [15], it is the ratio of the radiant energy from disk *i* reaching disk *j* to the whole radiant energy of disk *i*, as shown in Figure 4, and it can be calculated in Equation (9):

(9) $$F_{i-j}=\{h^{2}+r_{1}^{2}+r_{2}^{2}-\sqrt{{\left(h^{2}+r_{1}^{2}+r_{2}^{2}\right)}^{2}-4r_{1}^{2}r_{2}^{2}}\}/2r_{1}^{2}\;$$

$F_{di-dj}$ is the angle factor between ring *di* and ring *dj*, and $F_{di-j}$ ring *di* and disk *j*. Other angle factors can be deduced, assume $A_{i}$, $A_{di}$ are the areas of disk i and ring *di*, respectively, then $F_{i-dj}=F_{i-j}-F_{i-j+1}$, $F_{dj-i}=F_{i-dj}\times A_{j}/A_{d}{}_{i}$, $F_{dj-di}=F_{dj-i}-F_{dj-i+1}$, $F_{di-dj}=F_{dj-di}\times A_{dj}/A_{di}$, so the local effective emissivities along inside the wall of the cone, $\varepsilon_{a}(i)$ can be obtained as follows:

(10) $$\varepsilon_{a}(i)=\varepsilon +(1-\varepsilon )\sum_{j=1}^{N}\varepsilon_{a}(j)F_{di-dj}\;$$

**Figure 5:**
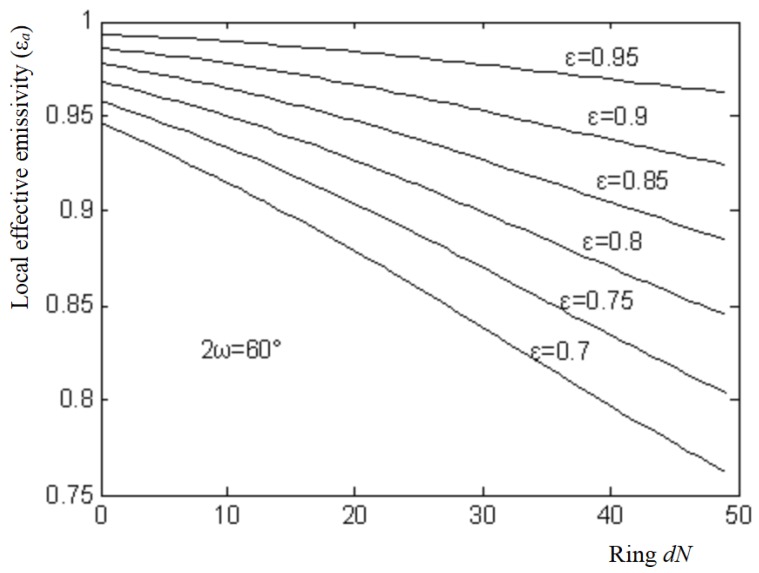
Local effective emissivity curves.

To solve Equation (10), the initial values of $\varepsilon_{a}(i)$ in Equation (10) are set to ε. An iteration process is performed. When the errors $\varepsilon_{a}(i)$ between two adjacent iterations are less than 10^−5^, the iteration process is stopped, and the convergence results $\varepsilon_{a}(i)$ are obtained. The results are as shown in Figure 5.

**Figure 6:**
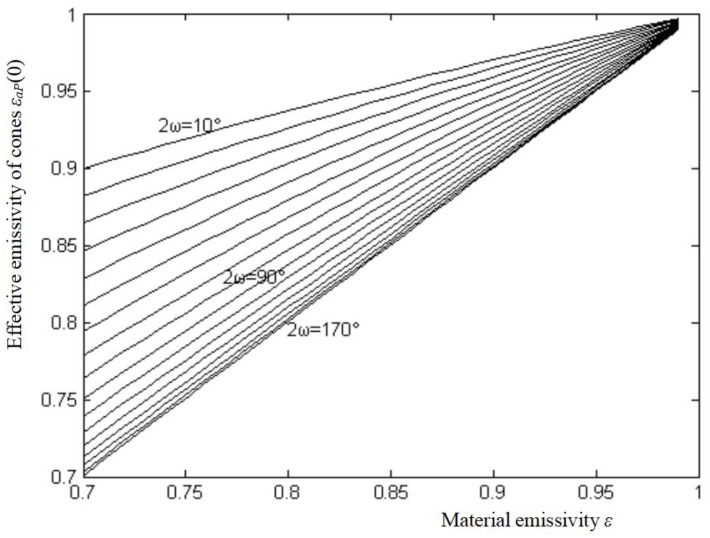
The effective emissivity of the cone.

The effective emissivities of the cone *ε_a_P*(0) can be calculated from Equation (11). Curves corresponding to cone angles 2ω reflect the changing of effective emissivities along with material emissivity *ε* which changes from 0.7~0.99 in 0.01 increments, and 2ω from 10°~170° in 10° increments, as shown in Figure 6.

(11) $$\varepsilon_{a}P(0)=\sum_{i=1}^{N}\varepsilon_{a}(i)A_{di}F_{di-N}\;$$

### 2.4. Comparison of the Two Methods’ Results

The exactitude methods such as the Bedford method are mostly used in blackbody radiant sources, but they are just suitable for sources with symmetric shapes such as a cone. As to the source with grooved surface, the Bedford method cannot be used, so Monte Carlo methods are developed. Monte Carlo methods are often deemed unstable and inaccurate, and their results fluctuate severely. The Monte Carlo method with large sampling number is expected to be more accurate. The effective emissivities of the cone *ε_a_P*(0) are calculated by both the Monte Carlo method and the Bedford method, respectively, along with material emissivity ε changing from 0.7~0.99 in 0.01 increments under the case when 2*ω* = 45°, and 60°.

**Figure 7:**
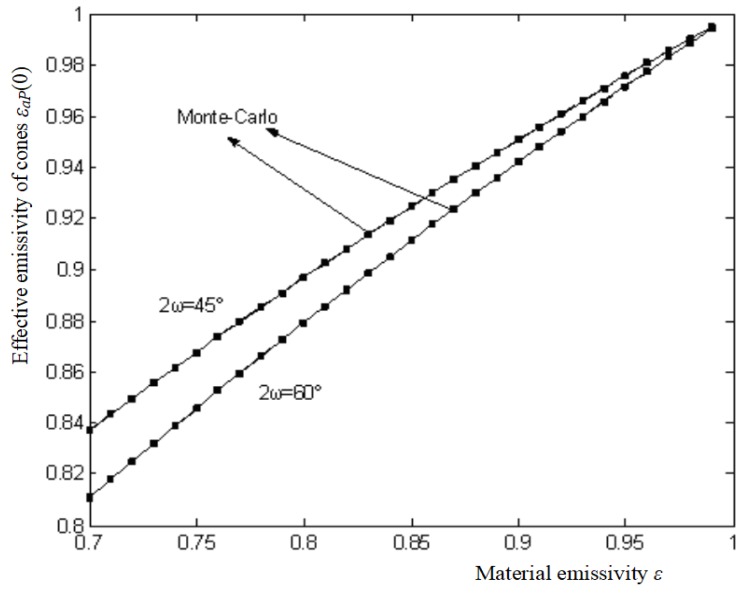
Comparison of the results between the two methods.

As an exactitude method, the calculation errors of the Bedford method are very small, within 10^−5^, but the Monte Carlo method is totally based on random testing, and although it can even be used for complex calculations, it is often regarded as a method with poor calculation precision. The Monte Carlo computing results show converging results require large sampling number (*N_s_*). The standard deviation is inversely proportional to $\sqrt{N_{s}}$. The results fluctuate severely when *N_s_* is less then 10^−6^, and they become stable when *N_s_* reaches 10^−7^. The Monte Carlo method needs very large samples, and for each calculation case, it needs 10^7^ samples (number of light points *N_s_*), and even up to 7 × 10^7^. Comparing the computation of the two methods, the *N_s_* of the Monte Carlo simulation is 2 × 10^7^. The results show that the maximum calculation error between the Monte Carlo and the Bedford method is limited to 0.0004, and the errors for most calculation points (the discrete dots represent the calculations by the Monte Carlo method) are usually around 0.0001~0.0002, that means the results from the both methods coincide (as shown in Figure 7). The Monte Carlo method is therefore also a credible method in thermal radiation analysis. By comparing the results both from the Monte Carlo method and the Bedford method, the accuracy of the Monte Carlo method is verified to be high enough, close to the Bedford method, so it is proper to use the Monte Carlo method to simulate the V grooved radiant surface. 

### 2.5. Monte Carlo Simulating for V-Grooved Surface

**Figure 8:**
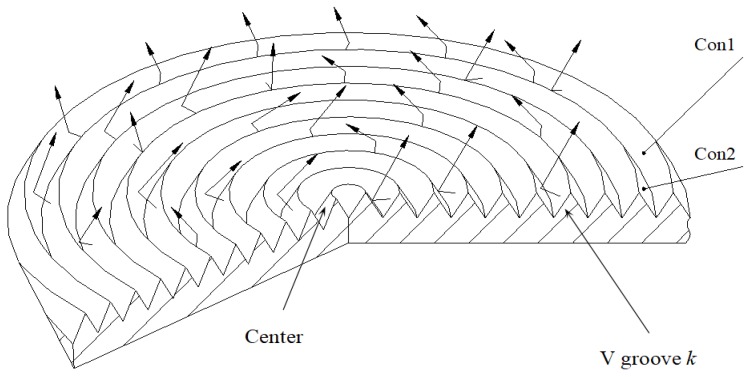
Surface with concentric V grooves.

To increase the effective emissivity, the radiant surface is processed into concentric V-shaped grooves, as shown in Figure 8. Because it is too difficult to use the exactitude numerical method, the Monte-Carlo method is employed to simulate the radiation of the surface. The radiant energy is regarded to be composed of independent beams emitted by light points which are evenly distributed on the surface. These beams are completely traced through the Monte Carlo simulation.

**Figure 9:**
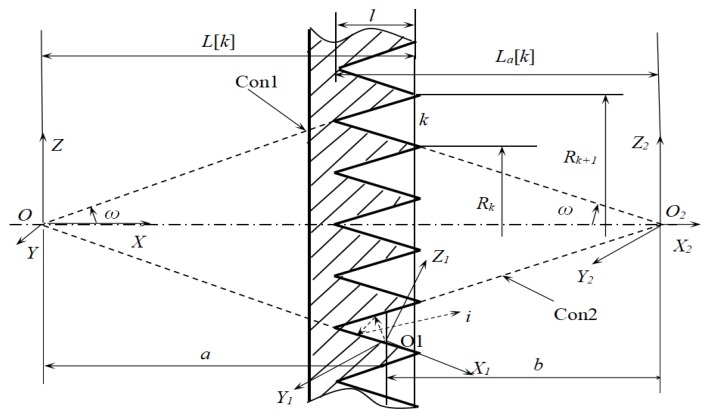
Light tracing for surface with concentric V-grooves.

To perform the Monte Carlo simulation, groove *k* is chosen, which is formed by two cones, the concave Con1 and the convex Con2, as shown in Figure 9. The first step is to determine the cone (Con1 or Con2) on which a light point is. When *r* ≤ *A_con_*_1_/(*A_con_*_1_ + *A_con_*_2_), the light point is on Con1, otherwise is on Con2. *A_con_*_1_, *A_con_*_2_ are the areas of the concave ring on Con1 and the convex ring on Con2 respectively. The second step is to determine the position of the light point. If it is on Con1, its position is *a*, if it is on Con2, its position is *b*. Both *a* and *b* are determined through random sampling as below:

(12) $$\begin{array}{l} a=L[k]-l+\frac{r(2L[k]-l)+3l-2L[k]}{2}\\ b=L_{a}[k]-l+\frac{r(2L_{a}[k]-l)+3l-2L_{a}[k]}{2} \end{array}$$

where *r* is a random number. The next step is to determine the direction (*θ*, *φ*) of a beam, and to trace the beam [16]. The tracing process is similar to the case for the cone. The emission or reflection of each beam of lights obeys the probability distributions. The Monte Carlo simulation process is to trace each beam of light until it is absorbed or ejected out of the groove. The angle of emission of the beam, whether the beam is absorbed or reflected at the point of reflection and the angle of reflection are all regarded as random events and are determined by random sampling. When the number of light points is large enough, the statistical result of effective emissivity of the groove will converge to the true value. When the simulation of all V-grooves on the surface is completed, the effective emissivity distribution of the surface is obtained.

**Figure 10:**
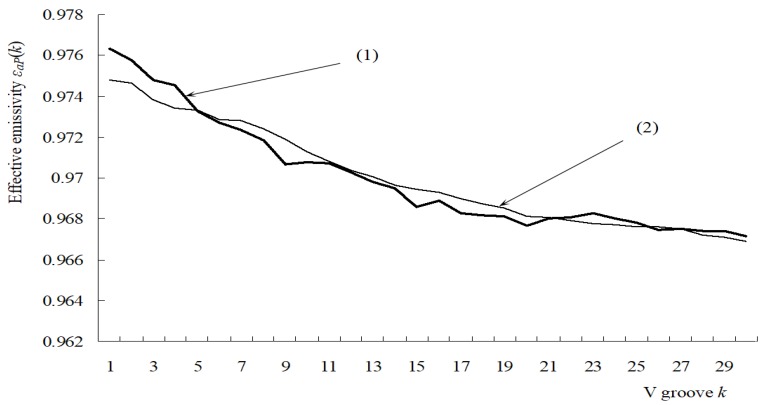
Distribution of effective emissivity of concentric V-groove surface.

The effective emissivity $\varepsilon_{a}P(k)$ of groove k is determined statistically after tracing all the rays. $\varepsilon_{a}P(k)=\varepsilon \cdot E_{out}\cdot (A_{con1}+A_{con2})/(A_{r}(k)\cdot N_{s})$, where $A_{r}(k)=\pi (R_{k+1}^{2}-R_{k}^{2})$ is the aperture area of groove k, *R_k+_*_1_, and *R_k_*, represent radii of two adjacent circles which forms the aperture of groove *k*. The Monte-Carlo program was performed under the Visual Studio.net2003 environment. The distribution of effective emissivity $\varepsilon_{a}P(k)$ of a round piece surface was obtained when all the grooves had been computed ($\varepsilon =0.95,\omega =22.5^{\circ }$, l=2.5). When the number of sampled light points is less than 5 × 10^6^, the results still fluctuate severely. The Monte Carlo calculation converges slowly [17], and when errors are less than 0.0002, the sampling number of light points is over 3 × 10^7^. From the simulation results, the values of $\varepsilon_{a}P(k)$ become lower slightly from the center to the edge. Curve (1) represents the results corresponding to the sampling number *N_s_* = 3.5 × 10^6^, and curve (2) is corresponding to *N_s_* = 3.5 × 10^7^, as shown in Figure 10.

**Figure 11:**
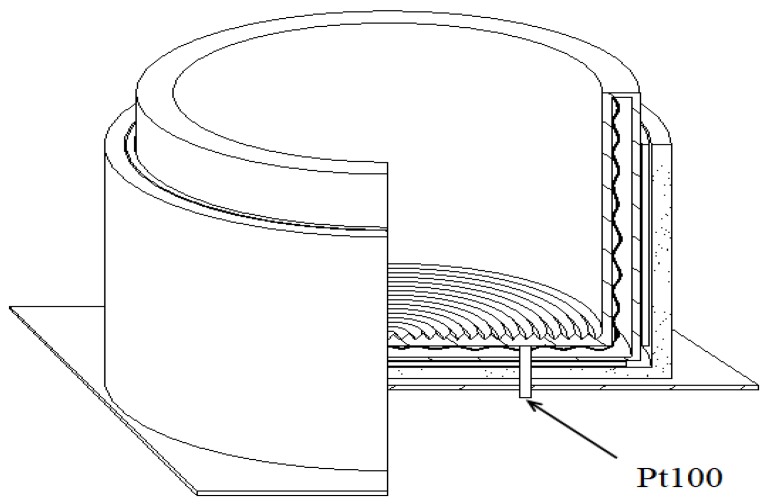
The structure of the radiant source.

The radiant source is composed of the V-grooved radiant surface, plus a cylinder to shield the circumference of the surface. The structure of the source is shown as in Figure 11. Heat pipe technology requires that the inside of the hollow shell is a capillary wicking porous material filled with heptane. This is an ideal area radiant source which can provide a uniform thermal radiation.

## 3. Control System Design

**Figure 12:**
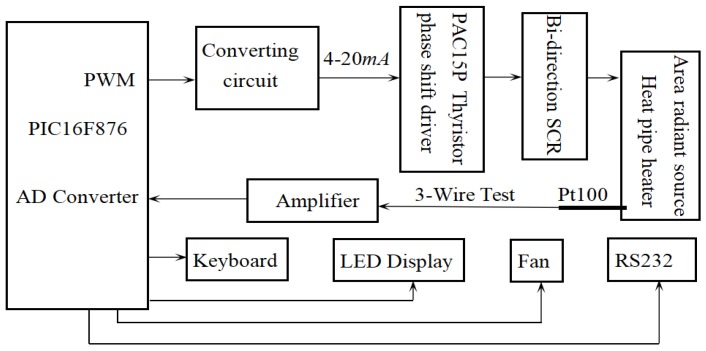
Control system with a PIC16F876 microprocessor.

The heating system possesses high heat inertia, and it is easily interfered by the environment. The fuzzy-PID control method is used in the temperature control system to make the system have a fast response, and operable across a large temperature range with high precision and strong robustness. The diagram of the control system is as shown in Figure 12. The primary task of the control system is to ensure the radiant source can reach a certain temperature in the temperature range of 5~95 °C. Heat pipe technology keeps the radiant source isothermal. Three Pt100 temperature sensors are installed on the heating surface to measure the temperature (as shown in Figure 11). A three-wire system is used to enhance the anti-interference ability. The temperature signals are amplified and transferred to a PIC16F876 microprocessor. The fuzzy-PID composite control method was adopted. The fuzzy control is appropriate for the large temperature range, and the PID is suitable to enhance the control precision [18]. The temperature signals are sampled by the A/D converter, and the control method decides the output. The power of the heater is controlled by a bidirectional silicon controllable rectifier (SCR) which is driven by the PWM outputs from the microprocessor. The incremental PID control is employed as shown in Equation (13) in which output values are decided by three adjacent sequential errors [19]:

(13) Δu(k)=Ae(k)−Be(k−1)+Ce(k−2)

where *A*, *B*, *C* are coefficients. *e* means error. *e*(*k*), *e*(*k* − 1), *e*(*k* − 1) are three sequential errors. All the control algorithms are based upon errors between the test value and the set value.

The fuzzy control method is widely used in temperature control systems [20], because it can imitate humans’ judgment. *E* denotes the temperature error, which is the error between the set value and the measured temperature value. *E_C_* represents the changes of temperature errors, and *U* denotes the fuzzy control outputs. Some symbols are used to represent the degree in a fuzzy set. NB denotes negative big, it expresses that deviation is negative big. NM negative medium, NS negative small, O zero, PS positive small, PM positive medium, and PB positive big. Assuming all the fuzzy variables’ domains of *E*, *E_C_* and *U* are {NB, NM, NS, O, PS, PM, PB}, the corresponding values are {−6, −5, −4, −3, −2, −1, 0, 1, 2, 3, 4, 5, 6}. Membership functions can be chosen as the triangle or Gaussian type, and the curves of the membership functions change along the fuzzy domain set. According to Zaden’s 21 condition sentences, the fuzzy inferences are performed. If the error *E* is equal to NB or NM, and the change of errors *E_C_* is NB or NM, then fuzzy output *U* should be PB under the fuzzy inference. The defuzzification of *U* can be got by Centroid method [18], as follows:

(14) $$z_{0}=\frac{\sum_{i=1}^{n}\mu_{c^{\prime }}(z_{i})\cdot z_{i}}{\sum_{i=1}^{n}\mu_{c^{\prime }}(z_{i})}\;$$

The digital values of output *U* are obtained through defuzzification under all conditions of *E*, *E_C_*. The output control table is formed by these values. Under a set of certain values of *E*, *E_C_*, the output *U* can be determined directly by looking up the output control table whose values can be got by using Matlab [17], the procedures are as follows:

(1) Using the fuzzy control tool box of Matlab, we enter the FIS editor window, and in the Edit menu, select FIS Properties item, and because the temperature fuzzy control system is a two-dimensional system, *E*, *E_C_* are chosen as input, and *U* is chosen as output.
(2) Editing the membership function of *E*, *E_C_* and *U*, selecting the discourse domain of *E* to be [−6, 6], selecting the membership function curves for the fuzzy subset {NB, NM, NS, ZO, PS, PM, PB} to be the Gaussian curve, and determining the gaussmf, parameter to be [0.8493, −2], and for *E_C_* and *U* the processes are similar.
(3) In the Edit menu, select the rules to edit, and the judging relationship between the two inputs *E*, *E_C_* is set as “and”. All fuzzy rule statements are entered. The fuzzy controller design is basically complete at this point, and the image corresponding to the input variables can be observed, and the process to determine the output variable value of the reasoning and the calculation process can be observed by the rule viewer, and through the surface viewer, the output surface map of *U* can be observed with the input *E*, *E_C_* changes.
(4) The control file is saved as TemperContol, and the digital values of *U* are placed in the control output table [18].

The control output table *U*[13][13] is the two-dimensional array whose capacity corresponds to the discourse domains of *E*, *E_C_*. The range of a PWM register output values is 0~1023. When the fuzzy control is performed, the fuzzy values of *E*, *E_C_* are used as the indexes of *U*[13][13], and the values of discourse domains of *E*, *E_C_* ([−6, 6]) should be converted into [0, 12].

The temperature control flow is as follows: when the difference between the measured value and the set value is greater than the error limit (which can be determined by adjustments), the system is controlled by the fuzzy control, otherwise, it is under PID control. In the fuzzy control, when *E* = −4, *E_C_* = 3, the output corresponds to *U*[2][9] (as to *E*, −4 is converted into 2, *E_C_*, 3 into 9), PWM output can be obtained by calling the function, set_pwm1_duty (*U*[2][9]).

## 4. Experiments

**Figure 13:**
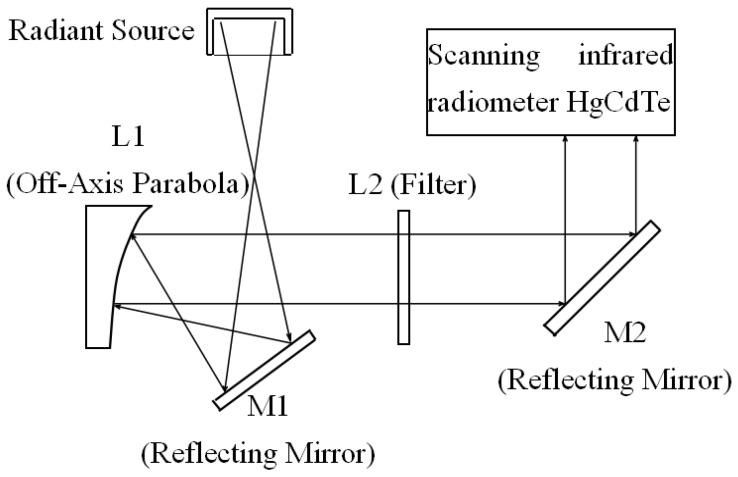
Optical test system for a radiant source.

**Figure 14:**
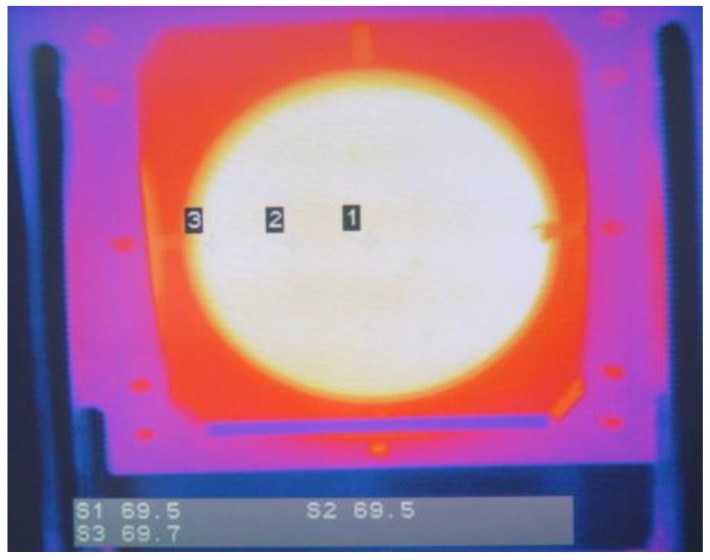
Thermal image of radiant source.

The radiation of this blackbody radiation source is experimentally verified by the optical system [21,22]. The radiation is confined to 8~12 μm far infrared light by the filter (see Figure 13), and then projected onto the HgCdTe infrared radiometer which performs a scanning test. Table 1 shows the results of the radiation field temperature under 40% RH laboratory humidity conditions. Figure 14 is a thermographic view of the scanning of the radiation source by the scanning radiometer. The standard deviation *S_D_* of the measurement results corresponding to each setting temperature is less than 0.05 °C. With the 95% confidence interval, the uniformity of measurement results which can be obtained from the radiation source is within ±0.1 °C. Because the radiant surface of the source is isothermal, the uniformity of the testing value of the scanning radiometer verifies the uniformity of the effective emissivity of the radiant surface, which is consistent with the theoretical analysis.

The radiation source was scanned every 5 min at the temperatures of 40 °C and 70 °C, and the temperature field stability of the radiation source was obtained by statistical calculation of the measurement results.

The Planck blackbody radiation law (Planck’s law) is the fundamental principle in infrared measurements. A blackbody’s temperature can be calculated by simply measuring the blackbody’s spectral emission power according to Planck’s law [23]. In recent years, multi-spectral temperature testing technology which is based on Planck’s law has been applied in practice. However, only radiometric parameters, such as radiance, are directly measured by the radiometer [24]. Through the corresponding test principle, the object radiation temperature can be deduced. In order to obtain the measured temperature, the emissivity of the measured object should be determined, but from another point of view, if the true temperature of the measured object is known, the performance of an infrared thermometer can be verified. The method of evaluating radiation source performance through radiation testing is presented here. If the temperature is determined by the two or more wavelengths of the specific temperature, the resulting color temperature of the object (*T_s_*) is obtained. If it is based on the temperature of an object radiation temperature, a characteristic wavelength of radiation temperature, then the measured object temperature is the luminous temperature (*T_l_*). An object radiation temperature, color temperature and luminous temperature are not the real surface temperature (*T*). When the real temperature of an object is *T*, its emissivity is *ε*(*T*), the radiant output of the object is *M*(*T*). When *M*(*T*) is equal to the radiation of a blackbody whose temperature is *T_τ_*, *T_τ_* is called the object radiation temperature:

(15) $$\varepsilon (T)\sigma T^{4}=\sigma T_{\tau }^{4}\;$$

when *ε*(*T*) is known, the real temperature of the object can be derived from the radiation temperature *T_τ_* according to Equation (15).

If the spectral emissivity of an object is *ε_λ_*(*T*), its spectral radiance is *L_λ_*(*T*). When *L_λ_*(*T*) is equal to the spectral intensity of a blackbody with temperature *T_l_*, *T_l_* is called the luminous temperature of the object. By the Planck’s law, the calculation of the real temperature is simplified in Equation (16):

(16) $$T=\frac{c_{2}T_{l}}{\lambda T_{l}\ln\varepsilon_{\lambda }(T)+c_{2}}\;$$

where *c*_1_ and *c*_2_ are the coeffients in the Planck’s law. $\varepsilon_{\lambda }(T)$ should be known in order to derive the real temperature of an object from the measured luminous temperature *T_l_*. Similarly, if the spectral emissivities under the wavelengths $\lambda_{1}$ and $\lambda_{2}$ are $\varepsilon_{\lambda_{1}}(T)$, $\varepsilon_{\lambda_{2}}(T)$, respectively, the real temperature *T* can be determined according to Equation (17):

(17) $$\frac{1}{T}-\frac{1}{T_{s}}=\frac{\ln[\varepsilon_{\lambda_{1}}(T)/\varepsilon_{\lambda_{2}}(T)]}{c_{2}(1/\lambda_{1}+1/\lambda_{2})}\;$$

where *T_s_* is the color temperature of the object. As to the measuring case, an object’s emissivity, the luminous temperature or the real temperature of the object can be obtained by comparing the standard blackbody’s temperature to the object radiation temperature [25]. If the emissivity of an object is known, its real temperature can be determined through comparing its radiation with a blackbody radiation. Conversely, if the real temperature of an object is known, its emissivity can be obtained [26]. Because the radiant source works in the wavelength band 8~12 μm, the following derivation is corresponding to a wavelength band. When the radiant source temperature is *T* and its spectral emissivity is ε(λ), the spectral luminance on a radiometer is as follows:

(18) $$E_{T}(\lambda )=\frac{1}{4}\varepsilon (\lambda )\tau_{a}(\lambda )\tau_{0}(\lambda )M_{0}(\lambda ,T){\left(\frac{D}{f^{\prime }}\right)}^{2}\;$$

where *D* and $f^{\prime }$ are the optical aperture and focal length of the optical system, $\tau_{a},\tau_{0}$ are the spectral transmittances of the atmosphere and the optical system respectively, *M*_0_ (*λ*, *T*) is a blackbody radiant flux. In the wavelength band *λ*_1_*~λ*_2_, the detection system output signal level is as below:

(19) $$U(T)=\frac{1}{4}A{\left(\frac{D}{f^{\prime }}\right)}^{2}\int_{\lambda_{1}}^{\lambda_{2}}R_{V}(\lambda )\varepsilon (\lambda )\tau_{a}(\lambda )\tau_{0}(\lambda )M_{0}(\lambda ,T)d\lambda \;$$

where, *R_V_*(*λ*) is the detector’s spectral response, and *A* is the area of the detector. When the test distance is fixed, the infrared transmittance is negligible. Assuming the average transmittance of the optical system in the infrared band *λ*_1_*~λ*_2_ to be $\bar{\tau }$, *R_V_*(*λ*) = 1 for an ideal detector, and the radiant source emissivity ε is independent of the wavelength, and the measured output level of the radiation source can be simplified as follows:

(20) $$U(T)=\frac{1}{4}A{\left(\frac{D}{f^{\prime }}\right)}^{2}\varepsilon \bar{\tau }\int_{\lambda_{1}}^{\lambda_{2}}M_{0}(\lambda ,T)d\lambda \;$$

Under the same test conditions, the output of the standard blackbody can be obtained. When the output level of the radiation source is equal to the standard blackbody output level, the emissivity of the radiation source is determined by the following equation:

(21) $$\varepsilon =\frac{\int_{\lambda_{1}}^{\lambda_{2}}M_{0}(\lambda ,T_{b})d\lambda }{\int_{\lambda_{1}}^{\lambda_{2}}M_{0}(\lambda ,T)d\lambda }\;$$

where, *T_b_* can be used as the measured radiation source wavelength band, by comparing with the standard radiation source to determine. *T* is the real temperature, measured by the temperature measurement circuit. The radiant flux of the blackbody source can be determined by Planck’s law, as follows:

(22) $$M_{0}(\lambda ,T)=\frac{\pi c_{1}}{\lambda^{5}[\exp(c_{2}/\lambda T)-1]}\;$$

Substituting Equation (22) into Equation (21), the emissivity ε of the radiation source at temperature *T* can be determined. In order to avoid the complexity of deriving the integral solution in Equation (22), the emissivity *ε* is calculated by programming. The integral domain is from *λ*_1_ to *λ*_2_ (*λ*_1_ = 8 μm, *λ*_2_ = 12 μm, and the integration step is *dλ* = 0.04 μm). Table 2 shows the test and calculation results.

The characteristics of a blackbody radiant source are often evaluated through theoretical analysis [27]. Theoretical evaluation on the effective emissivity has significance in the design of a radiant source. Material intrinsic emissivity ε is an important parameter in the evaluation of the emissivity of a radiant source, but it varies with the environmental conditions. This part is about the experimental research on the effective emissivity of the radiant source. The results of experimental tests show differences with the theoretical analysis of the effective emissivity. This is because that the theoretical analysis is based on the diffusion model in the whole spectrum band, and the practical measurements involve specular reflection in the 8~12 μm wavelength. There are differences between the actual situations and the ideal theoretical assumptions such as the isothermal and diffuse model, the size of the radiant source. As to the material intrinsic emissivity ε of the radiating surface, in the theoretical analysis, it is usually regarded as a constant, but in fact, ε itself also changes with the environmental temperature. If the effective emissivity of the radiation source calculated by theoretical analysis is adopted as the calibration parameter, it will inevitably produce large errors in the calibration of the actual infrared equipment, which will affect the infrared imaging quality [28]. From the analysis above, the uniformity of the emissivity of the radiation source should be verified by the uniformity of the radiation of the source in the testing spectrum band.

**Figure 15:**
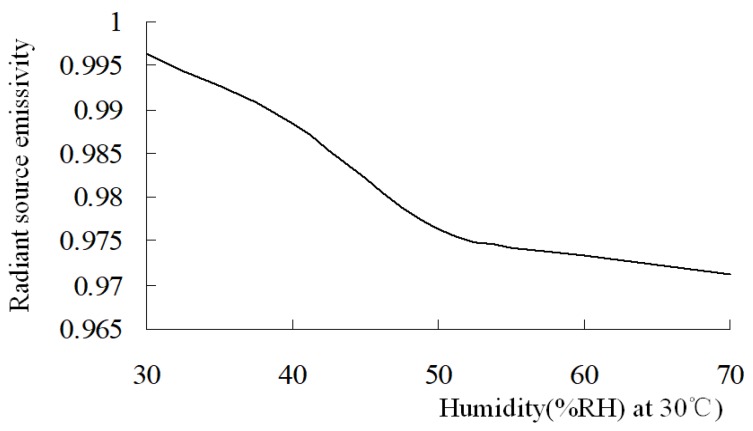
Calibration curve of the radiant source.

Fowler proposed the concept of black body mass (blackbody quality), and pointed out that the blackbody mass is dependent on wavelength and temperature, and it decreases with the increasing temperature [29,30]. The quality of blackbody is essentially a reflection of the radiation source, which is influenced by the radiant source structure, temperature, environment humidity, etc. This proves that humidity is sensitive to ultraviolet radiation [31]. The effective emissivity of a radiant source can be calibrated via a humidity sensor. After the calibration curve is obtained, it is easy to identify humidity by measuring the radiation of the radiant source. At a certain temperature, the emissivity of the radiation source should have some relationship with the changing environmental humidity. The calibration curve of relationship between the emissivity of the radiant source and humidity is obtained through calibration, which is implemented by the infrared radiant measuring process as the humidity changes at a temperature of 30 °C. Although the sensor DHT11 whose humidity testing precision is about ±5% RH suitable for 20~90% RH is not suitable to be used as for standard humidity measurements, here it is used just to show the radiance calibration method for humidity testing. The calibration curve for the emissivity of a radiant source changing with humidity is shown in Figure 15. The radiation measurement scheme is as shown in Figure 13, the data scanned by the radiometer were tested as the humidity changed and was measured by the DHT11. It can be found that the emissivity of the radiation source decreases rapidly with increasing humidity in the 30~50% RH range, and becomes smoothly downwards in the 50~70% RH range. Humidity can be measured by the radiation test on the radiant source, which possesses fast response and strong robustness compared with other methods. When the radiant source emissivity test value is 0.985, the humidity will be 43% RH with a test accuracy of ±5% RH, and ±1% RH resolution. The test precision is dependent on the calibration accuracy which is affected by the DHT11 humidity sensor calibration. If a highly precise humidity sensor such as the AM2301 were adopted, the testing accuracy would be improved. The advantage of the humidity test via the radiant source is its fast response. The humicap humidity sensor response time is usually more than 20 s, but the radiant humidity test takes 30 ms which is just the radiometer scanning time. 

## 5. Conclusions

The characteristics of a surface radiant source were analyzed by the Monte Carlo method which is a flexible method that can simulate any real problem no matter how complex it is, but only if can it be described probably. The correct Monte Carlo model which has guaranteed simulation correctness should be established. Its convergence rate is expected to be very slow. To obtain accurate results, a large number of samples should be sampled. From the Monte Carlo simulation results, the samples should be more than 2 × 10^7^. In addition, the random uniformity number and the correctness of the sampling method are also the important to obtain correct results. Besides the uniformity of random number and the reasonable model, the computation is also largely affected by the sampling number. The temperature control system of the radiant source has high thermal inertia and is susceptible to environmental temperature interference. The fuzzy-PID control method is incorporated with the heat pipe technology, which makes the system temperature be controlled at a high precision stable level with a rapid response. The characteristics of the radiant source were not only calculated theoretically, but also they were tested by a radiometer. The radiant spectrum band of the source was limited in the bandwidth of 8~12 μm via a lens filter. The results show that the temperature uniformity is within ±0.10 °C and the stability is within ±0.10 °C. A radiant source radiating out uniform energy can be used as a standard radiation source [18]. The radiant source can provide uniform thermal radiation over the radiant surface and radiate a stable radiant flux at a temperature in the range 5~95 °C, which can satisfy the demands for the calibration. Besides, the method of the calibration of the radiation of the radiant source is suitable to measure humidity, which is implemented by comparing the values of radiant flux which are obtained under both conditions of the laboratory humidity (e.g., 40% RH) and the real applied condition RH at the same temperature. Humidity measurements are easily affected by environmental factors such as temperature. Because humidity affects the radiation received by the radiometer, humidity can be determined indirectly by its radiation testing. Before performing a humidity test, a calibration is needed to obtain the calibration curve. The method of using a radiant source focuses on radiation measurement, which is just affected by humidity. The humidity testing accuracy of the method is largely dependent on the precision of the calibrating humidity sensor. Humidity sensors with humicaps often possess long response times, but the method which measures the radiation directly has a fast response. The method possesses advantages such as not being easily interfered by the environmental temperature, and a fast response. It is suitable for use in environmental humidity measurements in grain depots, greenhouses, warehouses, etc. The radiant source with a large radiant surface in the far infrared radiation region is therefore suitable for performing humidity tests at a near room temperature.

## Figures and Tables

**Table 1:** Tested Results of the radiant source radiation under 40% RH.

*T*	$\bar{T}$ (°C)	$\Delta T_{\max}$ (°C)	$S_{D}$ (°C)
30.00	29.87	0.32	±0.03
40.00	39.00	0.45	±0.04
50.00	48.30	0.41	±0.05
60.00	57.98	0.31	±0.04
70.00	67.66	0.29	±0.04

**Table 2:** Tested results of the radiant source emissivity.

*T_b_*	29.805	39.165	48.385	58.03	67.68
*T*	30.03	39.94	50.05	60.02	69.96
*ε*	0.9964	0.9884	0.9765	0.9735	0.9713
